# Editorial for the Special Issue “Bioactive Compounds of Natural Products on Metabolic Disorders and Complications”

**DOI:** 10.3390/cimb45070359

**Published:** 2023-07-05

**Authors:** Wai San Cheang

**Affiliations:** State Key Laboratory of Quality Research in Chinese Medicine, Institute of Chinese Medical Sciences, University of Macau, Macau SAR 999078, China; annacheang@um.edu.mo

Metabolic disorders are complex abnormalities involving impaired glucose and lipid metabolism, associated with complications such as kidney disease, cardiovascular disease, foot ulcer, retinopathy, and neuropathy. The therapeutic potential of natural products has drawn increasing attention. In this regard, Buljeta et al. published a comprehensive review of animal and human studies on the impact of red wine polyphenols on cardiovascular disease, diabetes, cancer, gut microbiota, and oral health [1]. Trebesova et al. also proposed that botanicals and plant bioactive compounds could potentially be a healthy supplement for the combined treatment of metabolic and anxiety disorders [2].

The authors of this Special Issue provide novel findings on the potential uses of natural products and responsible molecular mechanisms of metabolic disorders. Kim et al. demonstrate that the mixed herb extract of *Arum ternata*, *Poria cocos*, and *Zingiber officinale* showed stronger effects than single-herb to attenuate hyperlipidemia and associated hepatic steatosis in free-fatty-acid-induced HepG2 cells [3]. A methoxy derivative of resveratrol, 3,3′,4,5′-tetramethoxy-trans-stilbene, was shown to activate the insulin signaling pathway to ameliorate insulin resistance in high-glucose-induced HepG2 cells [4]. Esculetin, a compound derived from coumarin, inhibited oxidative stress and apoptosis in tert-butyl-hydroperoxide-stimulated HEK293 cells [5]. Based on the pharmacophore fragment of glitazars, Blokhin et al. synthesized a compound, isopimaric acid derivative as PPARα and PPARγ dual agonists, which exerted promising hypoglycemic and hypolipidemic activities in obese and diabetic mice [6].

Other authors have determined that the plasma levels of NO synthesis metabolites such as asymmetric dimethylarginie (ADMA) and citrulline are altered in children with chronic kidney disease (CKD), among which ADMA could be a potential marker of the gradual progression of CKD [7].

Inappropriate feeding in layer chickens results in excessive fat deposition and metabolic disorders, thereby affecting egg production. Considering the poultry industry, Liao et al. found that dietary supplementation with β-hydroxy-β-methylbutyrate could reduce abdominal lipid deposition in layer chickens through enhancing hepatic bile acid synthesis and gut microbiota function [8].

Many scientists from around the globe have contributed to this Special Issue of *CIMB*, titled “Bioactive Compounds of Natural Products on Metabolic Disorders and Complications”, resulting in the publication of eight papers reporting on potential diagnostic markers and therapeutic agents for metabolic disorders. Herein, the Guest Editor would like to thank all authors for their excellent contributions to this Special Issue.
